# Supplementary material for: A Global Screen for Assembly State Changes of the Mitotic Proteome by SEC-SWATH-MS
Source: Cell Syst. 2020 Feb 26;10(2):133–155.e6. doi: 10.1016/j.cels.2020.01.001 (PMC7042714; doi:10.1016/j.cels.2020.01.001)

Q9Y5K5 | UCHL5_HUMAN | UCHL5 UCH37 AD−019 CGI−70
Monomer MW [kDa]: 37.607 Monomer expected elution fraction: 49

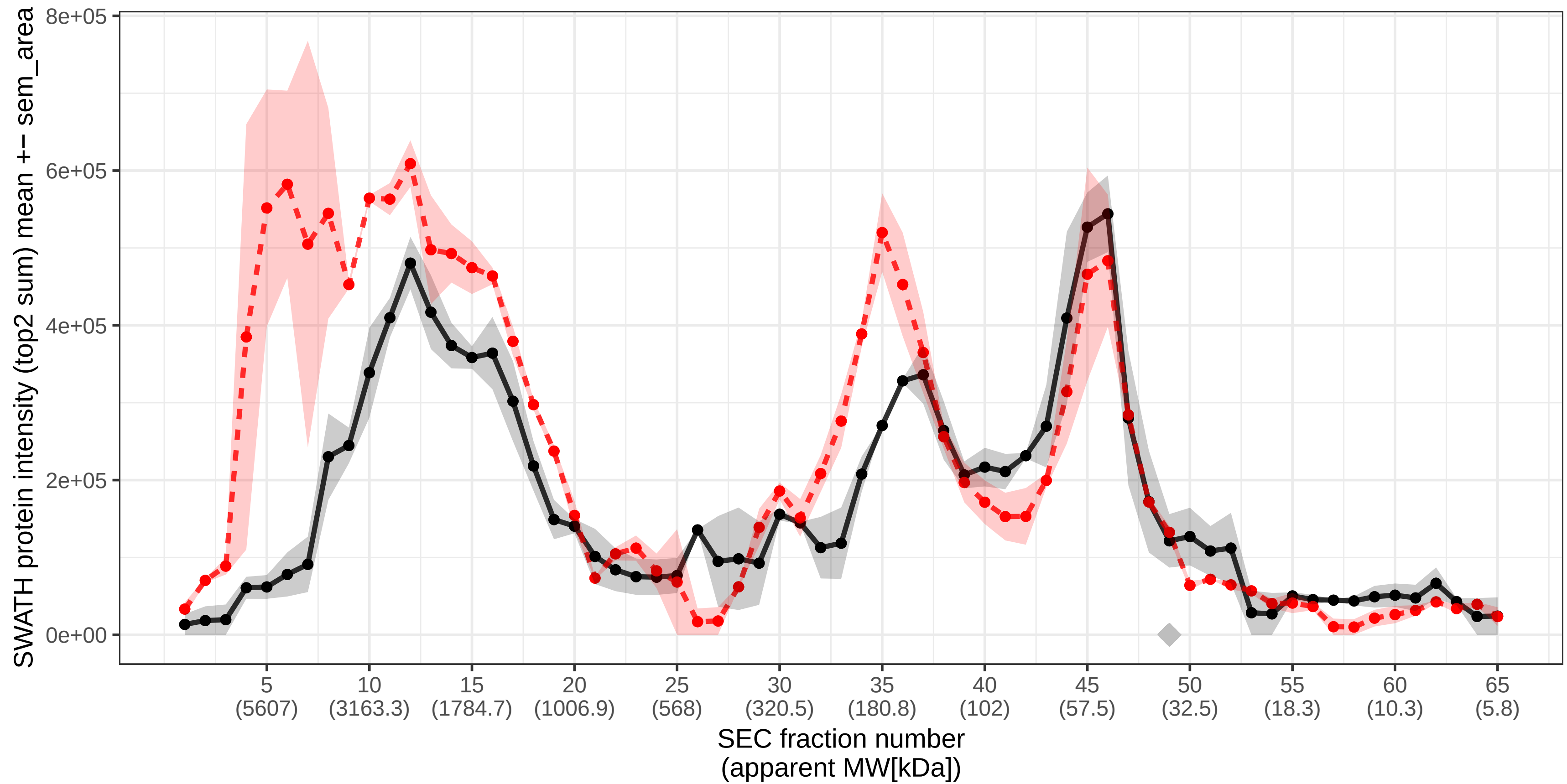

Supplement: Data S1. SEC-SWATH-MS Protein Chromatograms, Related to Figure 1 [file mmc6.zip › SECchrom_Q9Y5K5_UCHL5_HUMAN_UCHL5_UCH37_AD-019_CGI-70.pdf]
